# Neural Oscillations Underlying Guilt-Related Modulation of Visual Size Perception

**DOI:** 10.3390/bs16040541

**Published:** 2026-04-06

**Authors:** Ying Zhang, Mingyang Sun, Lihong Chen

**Affiliations:** 1 Institute of Psychological and Brain Sciences, Liaoning Normal University, Dalian 116029, China; zhangying_le89@163.com (Y.Z.); psysmy0323@163.com (M.S.); 2 Key Laboratory of Brain and Cognitive Neuroscience, Dalian 116029, China

**Keywords:** guilt, Ebbinghaus illusion, EEG, neural oscillations

## Abstract

Recent research demonstrates that guilt, as a self-conscious moral emotion, can shape early visual perception. However, the underlying neural mechanisms remain unclear. Using a pre–post experimental design combined with electroencephalography (EEG), we investigated how guilt modulates visual size perception and its neurophysiological correlates. Across four experiments, we confirmed that guilt emotion consistently increased the size overestimation component of the Ebbinghaus illusion. Time–frequency analyses revealed that guilt processing involved decreased prefrontal theta (4 to 7 Hz) power and reduced phase coupling of prefrontal theta and temporo-parieto-occipital alpha (8 to 12 Hz) oscillations. The guilt-related modulation of visual size perception was specifically associated with occipital alpha phase coherence. These results demonstrate that guilt emotion shapes fundamental visual processing through coordinated neural oscillations across large-scale brain networks. The findings advance understanding of emotion–cognition interactions and have implications for guilt-related psychiatric disorders.

## 1. Introduction

Guilt, as a self-conscious emotion, refers to the negative experience that arises when an individual’s behavior violates internalized moral standards (Amodio et al., 2007). Guilt is an exemplary moral and adaptive emotion, motivating prosocial behavior and strengthening social bonds. The feelings of guilt have been found to affect high-level cognitive abilities, such as impairing working memory performance (Cavalera et al., 2018) and biasing decision-making (Gangemi et al., 2025). Guilt also influences social attention, as demonstrated by enhanced facing-the-viewer bias (Shen et al., 2018) and reduced eye contact with the victims and gaze-cueing effect (Yu et al., 2017; W. Zhao et al., 2023). Recently, it has been found that guilt emotion can shape early visual perception as well. In particular, guilt relative to neutral emotion significantly increases the Ebbinghaus illusion effect, which is more pronounced for female than male participants (Sun & Chen, 2025). However, the underlying neural mechanisms behind this guilt-related modulation effect have not been well explored.

The prefrontal cortex plays a major role in top-down emotional control and guilt processing. For instance, patients with bilateral damage to the ventromedial prefrontal cortex donate significantly less in dictator games, suggesting insensitivity to guilt (Krajbich et al., 2009). Disruption of the left ventrolateral prefrontal cortex via repetitive TMS can alter the guilt-related modulation effect on visual size perception (Sun & Chen, 2025). Increased activation in prefrontal regions reduces both the intensity of negative emotional experiences and the activity of emotion-responsive brain regions (He et al., 2023; S. Li et al., 2024; Y. Li et al., 2025a; J. Zhao et al., 2021). Specifically, excitatory stimulation of the right dorsal lateral prefrontal cortex (dlPFC) strengthens top-down control of fearful stimulus processing, as reflected by reduced brain responses in the right occipital and temporal regions (Notzon et al., 2018). Inhibitory stimulation of the right prefrontal cortex elicits a stronger orienting response towards angry faces (d’Alfonso et al., 2000) and leads to stronger responses to fearful compared to neutral faces in temporal and parieto-occipital regions (Zwanzger et al., 2014). A single session of high-frequency repetitive TMS, using a 10 Hz stimulation protocol over the right dlPFC, produces a significant impairment in the ability to inhibit negative information (Leyman et al., 2009) and a significantly impaired disengagement from angry faces (De Raedt et al., 2010). Strengthened connections between frontal and parieto-occipital regions and greater similarity between the representation of the prefrontal cortex and the primary visual cortex have been observed during the processing of negative pictures as compared with neutral ones (Park et al., 2021; Yang et al., 2023). Taken together, the above findings suggest that the contribution of the prefrontal cortex to emotional control could possibly rest on its connections with parietal and occipital regions.

Converging evidence suggests that low-frequency oscillations in theta and alpha bands are associated with affective processing and its interactions with cognition. For instance, affective stimuli in comparison with neutral ones elicit greater theta oscillations at frontal sites (Aftanas et al., 2001, 2004; Balconi & Lucchiari, 2006; Balconi & Pozzoli, 2009). A neutral stimulus paired with a loud alarm elicits greater frontal theta activity in comparison with an unpaired one (Shner-Livne et al., 2025). A positive correlation between frontal theta activity and the valence of emotional pictures has been observed (Romeo & Spironelli, 2024). A study employing standardized low-resolution electromagnetic tomography implicated the left middle and inferior frontal gyri as likely generators of prefrontal theta oscillations during emotion regulation (Ertl et al., 2013). Research consistently shows that emotion regulation is associated with greater prefrontal theta oscillations compared to mere emotional experiences without regulation, with these increases positively predicting the effectiveness of emotion regulation (Domic-Siede et al., 2024; Ertl et al., 2013). Similarly, upregulating prefrontal theta oscillations via neurofeedback training can reduce subjective negativity ratings of negative stimuli (Y. Li et al., 2025b). Alpha band oscillations are predominantly localized to the parieto-occipital cortex (Clancy et al., 2022; Popov et al., 2019) and are involved in emotion–cognition interactions. It has been demonstrated that visual stimuli (i.e., gratings or color circles) paired with an aversive loud noise reduce posterior alpha power in comparison with unpaired stimuli (Bacigalupo & Luck, 2022; Friedl & Keil, 2020, 2021; Shner-Livne et al., 2025), and alpha suppression is observed in occipital regions contralateral to emotional distractors, and it correlates with impaired behavioral performance (Arana et al., 2022). Notably, it has been found that frontal theta–parietal alpha connectivity can predict speed effects during emotional interference (Mo et al., 2023).

The present study aimed to verify the reliance of the guilt-related modulation effect on basic visual size perception across a variety of experimental paradigms and investigate its underlying neural mechanisms by using the EEG technique. Feelings of guilt were established and associated with a specific face via a modified dots-estimation task. The magnitude of the Ebbinghaus illusion was measured both before and after the dots-estimation task in the three behavioral experiments, while it was measured only after the dots-estimation task in the EEG experiment. We expected that guilt emotion would enhance the size illusion magnitude, which would rely on emotional networks consisting of prefrontal, parietal and occipital regions and would be reflected by neural oscillations in specific frequency bands, such as theta and alpha.

## 2. Methods

### 2.1. Participants

A total of 98 female participants (mean age = 21.2 years, SD = 1.9 years) took part in the study, with 20 for Experiment 1 (10 for each group: guilt and neutral), 34 for Experiment 2 (17 for each group), 24 for Experiment 3 (12 for each group) and 20 for Experiment 4. All participants were female, as prior research has shown that the guilt-related modulation effect is reliably observed only in female participants (Sun & Chen, 2025). One of them was excluded due to excessive artifacts in Experiment 4. Sample sizes were determined based on previous relevant research (Sun & Chen, 2025). All participants had normal or corrected-to-normal eyesight and provided informed consent. They were naive to the purpose of the experiment. This study was approved by the institutional review board of Liaoning Normal University and was conducted in accordance with the principles of the Declaration of Helsinki.

### 2.2. Stimuli and Procedure

**Figure 1:**
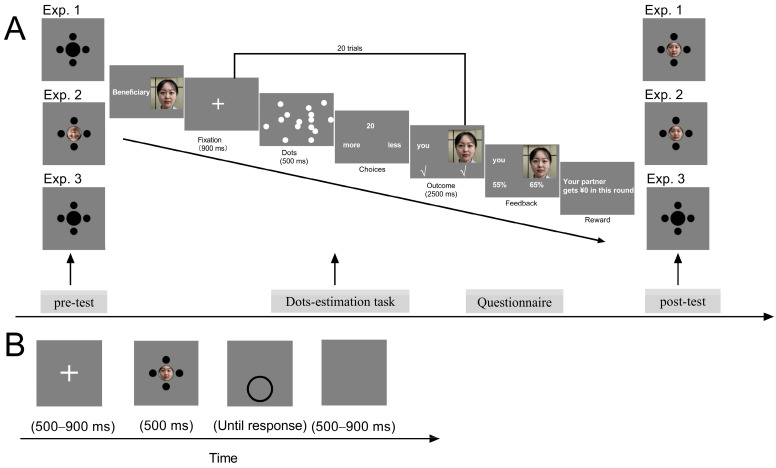
Schematic illustration of the experimental procedure. (**A**) Experiments 1, 2 and 3 adopted a pre–post design, in which the magnitude of the Ebbinghaus illusion was measured both before and after the dots-estimation task. During the size-matching task, participants were asked to adjust the size of a comparison circle to match that of the central target without a time limit. In the dots-estimation task, participants were required to perform a 2-round estimation of the dot number together with a partner. (**B**) Experiment 4 incorporated an EEG recording. The illusion effect was measured only after the dots-estimation task. During the size-matching task, an upright photo of the partner’s face was presented on the central target circle and was displayed for 500 ms, immediately followed by the presentation of the comparison circle.

Stimulus presentation was controlled using MATLAB R2022b (Mathworks, Natick, MA, USA) with Psychtoolbox extensions (Brainard, 1997; Pelli, 1997). We adopted stimuli and procedures similar to those in our previous study (Sun & Chen, 2025). A photo of a female face with a neutral expression was used to represent a simulated player. The Ebbinghaus illusion configuration consisted of a target circle (diameter = 1.1°) surrounded by four large or small circles (diameter = 1.7° and 0.6°, respectively). In the pre-test phase, the target circle was a black solid circle in Experiments 1 and 3, whereas an inverted face photo of the ostensible player was projected onto the target circle in Experiment 2. In the post-test phase, an upright photo of the ostensible player was projected onto the target circle for Experiments 1, 2 and 4, but the target circle was a black solid circle in Experiment 3 (Figure 1A). On each trial, the initial diameter of a comparison circle varied across 11 levels, ranging from 0.86° to 1.37° in 0.06° steps. The participants were positioned 57 cm from a gray computer screen (1920 × 1080 at 60 Hz), and their heads were stabilized with a chin rest.

Experiments 1, 2 and 3 used a pre–post between-subjects design. Specifically, the size-matching task was measured both before and after the dots-estimation task (Figure 1A). Half of the participants were in the guilt condition, and the other half were in the neutral condition. The dots-estimation task was the same for all four experiments. Experiment 4 employed a within-subjects design without the pre-test phase, where the participants performed the dots-estimation task twice on separate days, once under each condition (guilt or neutral). The size-matching task with the concurrent EEG recording was administered after the dots-estimation task.

#### 2.2.1. Dots-Estimation Task

This task was designed to create a context in which participants believed their performance directly affected the partner’s outcome, thereby eliciting guilt when their poor performance caused the partner to lose a bonus. Before the experiment, a confederate from our laboratory (the partner) entered the testing room, listened to the instructions together with the participant, and then left for another room under the pretense of performing tasks. The partner and the participants were strangers to each other. The participants were told that if both they and their partner achieved over 60% accuracy, one of them would receive a bonus. However, if either fell below 60%, neither would receive the bonus.

In the dots-estimation task, either the participant or the partner (indicated by a facial photograph) was designated as the bonus recipient at the start of the task. This photograph remained on the screen until the participant confirmed the information by pressing the space key. A fixation cross was then presented for 0.9 s, followed by 20 white dots appearing in random positions for 0.5 s. A reference number (19 or 20) subsequently appeared alongside the words “more” and “less”. The participants were instructed to judge whether the number of dots was greater or fewer than the reference number as quickly as possible.

Each round consisted of 20 trials. After each round, the accuracy of both the participant and the partner was displayed for 2.5 s, which was predetermined by the experimenter. Finally, feedback was presented indicating whether the participant or the partner earned or lost the bonus.

Guilt was manipulated via outcome feedback. The participants completed two rounds with their partners under either guilt or neutral conditions. The participant was the bonus recipient in Round 1, and the simulated partner was the recipient in Round 2. In the neutral condition, both earned the bonus for good performance. In the guilt condition, the participant earned the bonus in Round 1, but the partner lost the bonus in Round 2 due to the participant’s poor performance.

To assess changes in emotional states induced by the experimental manipulation, the participants rated their feelings immediately after the dots-estimation task. Using a 7-point scale (1 = not at all to 7 = very strongly), they evaluated six emotions, including sadness, shame, happiness, guilt, anger and pride, consistent with previous research (Zhu et al., 2019).

#### 2.2.2. Size-Matching Task

In Experiments 1, 2 and 3, a fixation cross was presented for 500 to 900 ms, then the Ebbinghaus configuration was presented at the screen center, with the comparison circle being simultaneously presented below it (8.6° from the screen center). The participants were required to adjust the size of the comparison circle to match that of the central target without a time limit. A total of 22 trials were administered, lasting approximately 4 min, with 11 trials for each condition (inducer size: large or small).

In Experiment 4, the Ebbinghaus configuration was displayed for 500 ms, followed by the presentation of the comparison circle (Figure 1B). The participants were required to adjust the size of the comparison circle to match that of the central target without a time limit. There was a total of 264 trials, with 66 repetitions for each experimental condition (inducer size: large and small; emotion: guilt and neutral). Each session, consisting of 132 trials, lasted approximately 20 min.

### 2.3. Data Recording and Preprocessing

In Experiment 4, EEG data were recorded from a scalp cap containing 32 electrodes using the international 10–20 system (Brain Products, Munich, Germany), with a sampling rate of 1000 Hz. The impedance was consistently maintained below 5 kΩ throughout the experiment. The data were preprocessed using EEGLAB (Delorme & Makeig, 2004) based on the MATLAB platform. EEG data were downsampled to 500 Hz, rereferenced to the average of TP9 and TP10, and filtered with a bandpass between 0.1 and 30 Hz. Blink and motion artifacts were corrected using an independent component analysis (ICA) algorithm. Noisy channels were identified by visual inspection and interpolated using a spherical spline interpolation method. Continuous recording was segmented into 3 s epochs. Baseline correction (−500 to 0 ms before stimulus onset) was applied to all epochs. After preprocessing, 57–59 epochs per condition remained (13% of epochs rejected).

For time–frequency analysis, data were segmented into 2 s epochs (−500 ms to 1500 ms) and were bandpass-filtered between 1 and 30 Hz. Time–frequency representations were computed for each participant using a short-time Fourier transform (STFT) with a fixed 300-ms Hanning window.

### 2.4. Data Analysis

#### 2.4.1. Behavioral Data Analysis

The magnitude of size overestimation (underestimation) was quantified as the percentage difference between the perceived size and the physical size of the central target when surrounded by small (large) inducers. To assess differences in illusion magnitude between guilt and neutral conditions, we conducted repeated measures ANOVA, along with independent and paired samples *t*-tests (two-tailed).

#### 2.4.2. Time–Frequency Analysis

Time–frequency decomposition was used to isolate the amplitude and the phase of the signal at each time and frequency. Signal power was computed by squaring the magnitude of the STFT coefficients, representing the energy density distribution across time–frequency bins. Inter-trial phase coherence (ITC) was quantified to assess phase-locking consistency, with values bounded between 0 (random phase distribution) and 1 (perfect phase alignment). For each trial, instantaneous phases were extracted from the complex STFT output, and ITC was derived as the normalized vector length of the circular mean phase across trials. False Discovery Rate Correction (FDR) was performed for multiple comparisons. The analysis of neural oscillations focused on theta (4 to 7 Hz) and alpha (8 to 12 Hz) oscillations.

#### 2.4.3. Phase–Phase Coupling (PPC)

PPC is an index used to quantify the phase relationships between two EEG signals, particularly suitable for analyzing the phase synchrony of neural oscillations (Belluscio et al., 2012). The PPC metric is bounded between 0 and 1, where 0 denotes statistically independent phase relationships between frequency bands, suggesting a uniform circular distribution of phase differences, and 1 reflects perfect phase synchrony, indicating a rigid phase-locking relationship across oscillations (Vinck et al., 2010).

Based on the results of time–frequency analysis, pairwise phase consistency values (i.e., PPC) were computed for theta (4 to 7 Hz) and alpha (8 to 12 Hz) frequency bands across experimental conditions. PPC values were assessed both within individual electrodes and between distinct electrode pairs. Specifically, we calculated phase differences between frontal theta oscillations and temporo-parieto-occipital alpha oscillations. Statistical comparisons between guilt and neutral conditions were conducted using paired sample *t*-tests. Pearson correlation analysis was performed to measure the relationship between PPC values and behavioral performance. All statistical results were corrected for multiple comparisons using the FDR method.

## 3. Results

### 3.1. Experiment 1

In Experiment 1, the central target was a black solid circle in the pre-test phase, which was an upright photo of the ostensible player in the post-test phase. For the dots-estimation task, the participants reported higher levels of guilt in the guilt condition relative to the neutral condition (mean difference = 2.8, *t*(18) = 4.16, *p* < 0.001, *d* = 1.86), indicating that the experimental manipulation was successful. Moreover, in the guilt condition, the ratings of guilt were significantly higher than the ratings of other emotions (*p*s < 0.05; Table 1).

**Figure 2:**
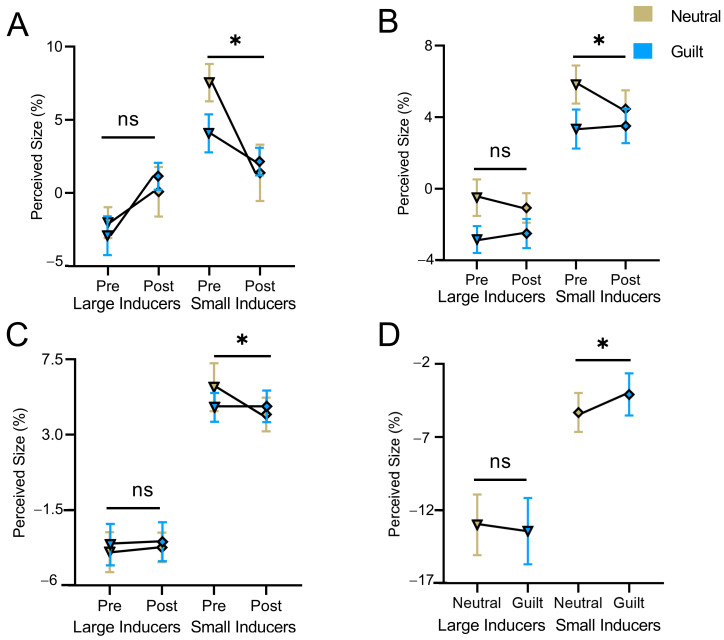
Behavioral results. Perceived size of the central target as a function of test time (pre and post), inducer size (large and small) and emotion (guilt and neutral) in (**A**) Experiment 1, (**B**) Experiment 2 and (**C**) Experiment 3. (**D**) Perceived size of the target as a function of emotion and inducer size for the first half trials in Experiment 4. Error bars represent one standard error of the mean. Asterisk (*) indicates significance level of * *p* < 0.05, and ns indicates no statistical significance.

For the size-matching task, when the inducer size was small (i.e., size overestimation), the main effect of test time (pre vs. post) was significant (*F*(1,18) = 34.63, *p* < 0.001, $\eta_{p}^{2}$ = 0.69), with size overestimation being smaller in the post-test phase than the pre-test phase. The main effect of emotion (guilt vs. neutral) was not significant (*F*(1,18) = 0.55, *p* = 0.467, $\eta_{p}^{2}$ = 0.03). The interaction between test time and emotion was significant (*F*(1,18) = 9.45, *p* = 0.007, $\eta_{p}^{2}$ = 0.34; Figure 2A). Further analysis showed that size overestimation was significantly decreased in the post-test phase for neutral emotion (*t*(9) = 6.15, *p* < 0.001, *d* = 1.94), which might be caused by the practice effect. However, for guilt emotion, though a similar pattern of results was observed, it failed to reach significance (*t*(9) = 2.05, *p* = 0.070, *d* = 0.65). We did not observe significant results for size underestimation and the overall illusion magnitude (i.e., the disparity of overestimation and underestimation; *p*s > 0.06).

### 3.2. Experiment 2

To control for potential confounding of stimulus feature differences between the pre-test and post-test phases, Experiment 2 employed an inverted face in the pre-test phase and an upright face in the post-test phase. The dots-estimation task and the procedure were the same as those in Experiment 1.

For the dots-estimation task, the participants reported higher levels of guilt in the guilt condition relative to the neutral condition (mean difference = 4.0, *t*(32) = 9.48, *p* < 0.001, *d* = 3.25), and the ratings of guilt were significantly higher than the ratings of other emotions in the guilt condition (*p*s < 0.05).

For the size-matching task, when the inducer size was small, the main effects of test time and emotion were not significant (*p*s > 0.09). The interaction between test time and emotion was significant (*F*(1,32) = 4.89, *p* = 0.034, $\eta_{p}^{2}$ = 0.13; Figure 2B). The results revealed that there was a decrease in size overestimation in the post-test phase relative to the pre-test phase for the neutral condition, whereas the opposite pattern of results was observed for the guilt condition. We did not observe significant results for size underestimation and the overall illusion magnitude (*p*s > 0.20).

### 3.3. Experiment 3

In Experiment 3, the central target was a black solid circle in both the pre-test and post-test phases. The dots-estimation task and the procedure were the same as those in Experiment 1.

For the dots-estimation task, similar patterns of results were observed as those in Experiments 1 and 2. Specifically, the participants reported higher levels of guilt in the guilt condition relative to the neutral condition (mean difference = 2.9, *t*(22) = 11.39, *p* < 0.001, *d* = 4.65), and the ratings of guilt were significantly higher than the ratings of other emotions in the guilt condition (*p*s ≤ 0.05).

For the size-matching task, when the inducer size was small, the main effects of test time and emotion were not significant (*p*s > 0.05). The interaction between test time and emotion was significant (*F*(1,22) = 4.64, *p* = 0.042, $\eta_{p}^{2}$ = 0.17; Figure 2C). Further analysis revealed that there was a decrease in size overestimation in the post-test phase relative to the pre-test phase for the neutral condition, whereas the opposite pattern of results was observed for the guilt condition. We did not observe significant results for size underestimation and the overall illusion magnitude (*p*s > 0.07).

### 3.4. Experiment 4

#### 3.4.1. Behavioral Results

For the dots-estimation task, the participants reported higher levels of guilt in the guilt condition relative to the neutral condition (mean difference = 3.8, *t*(18) = 9.11, *p* < 0.001, *d* = 1.81), and the ratings of guilt were significantly higher than the ratings of other emotions in the guilt condition (*p*s < 0.05).

For the size-matching task, as the guilt-related modulation effect might be short-lasting, we split all trials (*N* = 264) into two halves. For the first-half trials, the results showed that guilt emotion significantly increased size overestimation compared with neutral emotion (*t*(18) = 2.23, *p* = 0.039, *d* = 0.51; Figure 2D), and a similar pattern of results was observed for the overall illusion effect (*t*(18) = 2.21, *p* = 0.040, *d* = 0.50). However, this guilt-related enhancement was not observed for size underestimation (*t*(18) = −0.51, *p* = 0.620, *d* = 0.12). For the second-half trials, neither the main effects nor their interaction was significant (*p*s > 0.60).

#### 3.4.2. Time–Frequency Results

**Figure 3:**
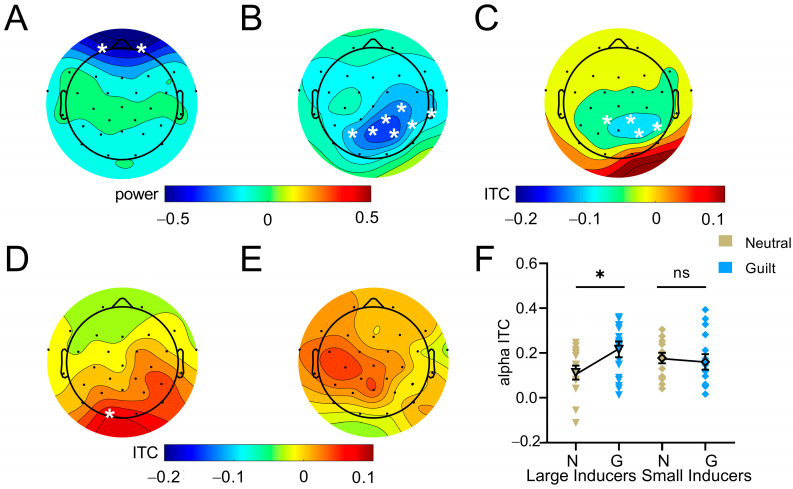
Results of time–frequency analysis. (**A**) Topographic distribution of theta power differences between guilt and neutral conditions. Topography of (**B**) alpha power and (**C**) alpha ITC disparity of large- and small-inducer conditions. Topography of alpha ITC difference between guilt and neutral conditions during the post-test phase for (**D**) size underestimation and (**E**) size overestimation. (**F**) Alpha ITC plotted as a function of emotion and inducer size. Error bars represent one standard error of the mean. Asterisk (*) indicates significance level of * *p* < 0.05, and ns indicates no statistical significance.

Repeated measures ANOVA showed that the main effect of emotion (guilt vs. neutral) was manifested by decreased theta power over frontal areas (FP1 and FP2) in the early time window (50 ms to 150 ms after illusion onset; *p*s < 0.05, uncorrected; Figure 3A). The main effect of inducer size (large vs. small) was demonstrated by decreased alpha power (150 ms to 250 ms; C4, CP2, CP6, T8, PZ, P4, and P3; *p*s < 0.05, FDR-corrected; Figure 3B) and alpha ITC (100 ms to 200 ms; CP1, CP2, CP6, and P4; *p*s < 0.05, FDR-corrected; Figure 3C) over central parietal regions in the early post-stimulus time window. The interaction between emotion and inducer size was revealed by occipital alpha ITC in the late post-stimulus time window (300 ms to 400 ms; O1: (*F*(1,18) = 14.61, *p* = 0.001, $\eta_{p}^{2}$ = 0.45, FDR-corrected; Figure 3D,E). Further analysis showed that the guilt condition relative to the neutral condition significantly increased alpha ITC for size underestimation (*t*(18) = 3.00, *p* = 0.046, *d* = 0.76; Figure 3F) instead of size overestimation (*t*(18) = −0.50, *p* = 0.872, *d* = 0.13).

#### 3.4.3. Results of Phase–Phase Coupling

We analyzed PPC values of theta and alpha oscillations at the same electrodes, as well as those of theta oscillations at frontal electrodes (FP1 and FP2) and alpha oscillations at temporo-parieto-occipital electrodes.

**Figure 4:**
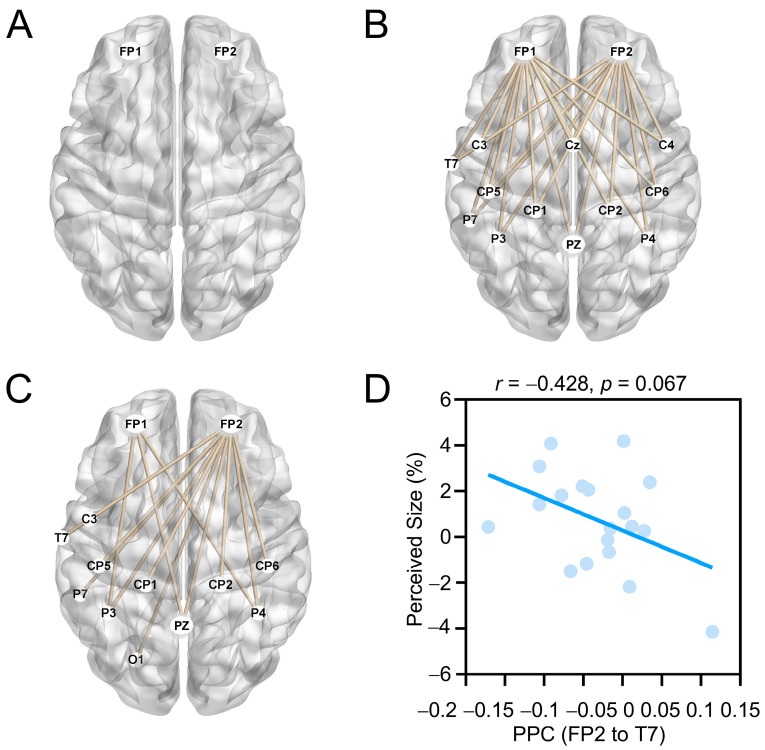
PPC results. Guilt relative to neutral condition significantly reduced (**A**) frontal theta–alpha phase coupling at individual electrodes (FP1 and FP2) and decreased inter-regional phase coherence between frontal theta and temporo-parieto-occipital alpha oscillations in both (**B**) size underestimation and (**C**) size overestimation conditions. (**D**) Correction between the phase coherence of frontal theta–temporal alpha (FP2 to T7) and perceived size overestimation.

For size underestimation, the results of paired sample *t*-tests (guilt vs. neutral, *p* < 0.05, FDR corrected) revealed significantly decreased frontal theta–alpha phase coupling at single electrodes (FP1 and FP2; 50 ms to 250 ms after illusion onset; Figure 4A) and decreased inter-regional phase coherence of frontal theta and temporo-parietal alpha oscillations (200 ms to 300 ms after illusion onset; Figure 4B). For size overestimation, a similar pattern of results was observed. Specifically, guilt relative to neutral emotion significantly decreased PPC values over frontal theta and temporo-parieto-occipital alpha oscillations (200 ms to 300 ms after illusion onset; Figure 4C). Correlation analysis showed that phase coherence of frontal theta and temporal alpha oscillations (FP2 to T7) demonstrated a trend to predict the strength of size overestimation when the neutral condition was subtracted from the guilt condition (*r*(19) = −0.428, *p* = 0.067; Figure 4D).

## 4. Discussion

By using a pre–post design with the victim’s face projected onto the target circle of the Ebbinghaus illusion during the post-test phase, the present study showed that, compared to neutral emotion, guilt emotion significantly increased the size overestimation component of the Ebbinghaus illusion, while no guilt-related regulation was observed for size underestimation (Experiment 1). These findings were replicated when the inverted face of the victim was projected onto the target circle during the pre-test phase (Experiment 2) and when the victim’s face was absent from both the pre-test and post-test phases (Experiment 3), as well as when employing a within-subjects post-test design (Experiment 4). Neurophysiological analysis revealed that the main effect of emotion (guilt vs. neutral) was demonstrated by attenuated frontal theta power in the early time window and decreased phase coupling of frontal theta and temporo-parieto-occipital alpha oscillations in a relatively late time window. The interaction between emotion and inducer size was revealed by occipital alpha ITC in the late time window, with guilt emotion relative to neutral emotion enhancing alpha phase coherence in size underestimation rather than overestimation.

Previous research on affective modulation of visual size perception has predominantly examined basic emotions, such as fear and anger. These studies demonstrate that individuals tend to overestimate the size of fearful stimuli and neutral stimuli when experiencing fear (Chen et al., 2016; Leibovich et al., 2016; Lourenco et al., 2011; Shiban et al., 2016; Vasey et al., 2012), and prior exposure to fearful primes can significantly decrease the magnitude of the Ebbinghaus illusion (Hu et al., 2023). Similarly, when negative images are presented on the central target of the Ebbinghaus configuration, the illusion effect is substantially reduced (van Ulzen et al., 2008). In contrast to basic emotions, negative moral emotions produce opposing effects on visual size perception. Specifically, when a victim’s face is projected onto the central target of the Ebbinghaus configuration, the illusion strength is significantly increased (Sun & Chen, 2025). The present study is consistent with these findings, showing that guilt emotion enhances the size overestimation component of the Ebbinghaus illusion in both pre–post and post-test designs, even when no direct associations exist between guilt emotion and size perception. Compared to fear emotion, guilt emotion emerged later in our evolutionary history and is considered cognition-dependent, requiring a high level of conscious self-awareness and self-representation (Gilead et al., 2016). Accordingly, we conjecture that during guilt states, participants might expend greater cognitive resources on either suppressing guilt experiences from conscious awareness or engaging in social-evaluative processing, thereby leaving less attentional capacity for processing external stimuli, resulting in increased contextual modulation, as reflected in the enhanced illusion effect.

A distributed neural network consisting of prefrontal, temporal, parietal and occipital regions is associated with guilt processing. A meta-analysis of neuroimaging studies showed that guilt is linked to the anterior cingulate gyrus, the superior and medial frontal gyri, the superior temporal gyrus, the parietal cortex, and the occipital cortex (Gifuni et al., 2017). Guilt relative to neutral emotion elicits greater activations in the anterior cingulate, the insular, inferior and medial prefrontal cortices, and the anterior and superior temporal cortex (Shin et al., 2000; Takahashi et al., 2004). Consistent with and extending these findings, the present study reveals that guilt relative to neutral emotion decreases prefrontal theta activity and attenuates phase synchronization of frontal theta and temporo-parieto-occipital alpha oscillations. Decreased theta power and reduced phase synchronization have been observed during successful suppression of unwanted memories (Legrand et al., 2020; Waldhauser et al., 2015). Moreover, a transient reduction in theta activity is observed with an attentional shift (Ohgomori et al., 2025). These parallels suggest that the attenuated frontal theta power and phase synchronization observed in the present study might reflect active suppression of guilt-related experiences, likely by shifting attention away from the victim.

The contribution of alpha oscillations to emotion–cognition interactions is well-documented. Visual stimuli associated with affective attributes can attenuate alpha power in parieto-occipital regions (Bacigalupo & Luck, 2022; De Cesarei & Codispoti, 2011; Friedl & Keil, 2020, 2021; Shner-Livne et al., 2025). The present study reveals that guilt-related modulation of visual size perception rests on occipital alpha activity. Specifically, guilt emotion, compared to neutral emotion, induces greater alpha phase coherence in size underestimation instead of size overestimation. It should be noted that guilt emotion strengthens perceived size overestimation rather than underestimation at the behavioral level. Alpha activity has been associated with the suppression of distracting information, as demonstrated by stronger power increase and phase coherence in temporo-occipital areas prior to strong versus weak distractor onsets (Bonnefond & Jensen, 2012). Thus, we propose that during size underestimation trials, participants exert greater cognitive effort to inhibit guilt feelings, as reflected in increased alpha phase coherence. This enhanced inhibitory processing might effectively reduce guilt-related interference, thereby reducing the guilt-related modulation effect on behavior performance in the size underestimation condition. Conversely, the relatively lower alpha coherence during size overestimation trials might reflect weaker suppression of guilt experiences, allowing guilt to exert a stronger modulatory influence on size perception, as evidenced by the enhanced illusion effect. This conjecture is indirectly supported by the temporally distinct neural signatures of guilt emotion, as demonstrated by an early reduction in frontal theta power (50–150 ms) preceding a later modulation of occipital alpha activity (300–400 ms) linked to visual size perception.

The present study has several limitations that warrant consideration. The interpretation of Experiments 1–3 is constrained by an alternative explanation that guilt might impede task learning, not solely counteract practice effects. Furthermore, the dissociation between neural (alpha ITC enhancement for large inducers) and behavioral (illusion increase for small inducers) outcomes reveals a complex, context-dependent mechanism, not a direct linear relationship. Future studies should employ alternative experimental inductions to isolate the specific effects of guilt from the influence of other co-occurring emotions, record response time during size illusion tasks and investigate whether the observed guilt-related effects can generalize across genders or are modulated by gender-specific factors.

Taken together, the present study demonstrates that the experience of guilt enhances the Ebbinghaus illusion across multiple experimental paradigms and further reveals that the guilt-related modulation effect on visual size perception is associated with occipital alpha activity. The findings indicate that guilt feelings not only shape high-level prosocial behavior but also alter fundamental visual processing. This work provides new insights into the mechanisms of emotion–cognition interactions and has implications for understanding guilt-related disorders, such as obsessive–compulsive disorder and depression.

## Figures and Tables

**Table 1:** Ratings of emotional states in the dots-estimation task (M(SD); 1 = not at all, 7 = very strongly). Asterisk (*) indicates significance level of * *p* < 0.05, ** *p* < 0.001.

Emotion	Experiment 1			Experiment 2			Experiment 3			Experiment 4		
	Guilt	Neutral	*t*	Guilt	Neutral	*t*	Guilt	Neutral	*t*	Guilt	Neutral	*t*
Guilt	5.4(1.4)	3.0(2.0)	4.16 **	5.4(1.3)	2.6(1.7)	9.48 **	5.5(1.6)	1.5(0.7)	11.39 **	5.6(1.3)	1.8(1.2)	9.11 **
Shame	4.3(2.1)	2.0(1.0)	2.83 *	4.3(2.1)	2.2(1.1)	7.26 **	4.8(1.3)	1.9(1.1)	4.84 **	4.0(1.6)	2.0(1.3)	4.30 **
Anger	2.2(0.8)	2.0(1.0)	1.20	2.2(0.8)	1.7(1.1)	2.02	2.1(1.0)	1.5(0.7)	2.64 *	1.8(1.0)	1.4(0.7)	2.67 *
Pride	1.8(1.0)	5.0(2.0)	−4.01 **	1.8(1.0)	4.6(2.0)	−3.68 **	2.3(1.1)	3.9(1.5)	−3.69 **	2.2(1.5)	4.3(1.5)	−4.71 **
Sadness	4.2(1.8)	1.0(1.0)	4.70 **	4.2(1.8)	1.4(0.7)	4.53 **	3.5(1.4)	1.7(0.9)	3.55 *	3.8(1.6)	1.6(1.1)	6.27 **
Happiness	2.4(1.3)	5.0(2.0)	−4.01 **	2.4(1.3)	5.2(1.8)	−3.66 **	3.2(1.2)	4.9(1.6)	−4.11 **	2.8(1.3)	5.4(1.2)	−7.30 **

## Data Availability

All data supporting the findings of this study are available from the corresponding author upon request due to privacy and confidentiality agreements with the participants.
